# Supplementary material for: Benchmarking the PEPOP methods for mimicking discontinuous epitopes
Source: BMC Bioinformatics. 2019 Dec 30;20:738. doi: 10.1186/s12859-019-3189-3 (PMC6937815; doi:10.1186/s12859-019-3189-3)
Supplement: Supplementary file 2 — Additional file 2: Figure S1. Description of the prime, ALA linker, structural alphabet linker and structural alphabet superposition linker methods. Figure S2. Sequence redundancy between peptides predicted by the various PEPOP methods. Figure S3. Characterization of PEPOP clusters and patches. Figure S4. Mean Se (A) and PPV (B) by method. Empty bars: results that did not take into account the aa positions; filled bars: results taking into account the aa positions. Figure S5. Se and PPV distribution without taking into account the positions of the peptides predicted by A) the NN methods (NN, NNala, NNsa, NNsas, uNN); B) the FN methods (FN, FNala, FNsa, FNsas); C) the ONN methods (ONN, ONNala, ONNsa, ONNsas); D) the OPP methods (OPP, OPPala, OPPsa, OPPsas); E) the SHP methods (SHPnat, SHPrev, SHPaa); and F) the TSP methods (TSPnat1, TSPnat2, TSPnat3, TSPnat4, TSPrev1, TSPrev2, TSPrev3, TSPrev4, TSPaa). [file 12859_2019_3189_MOESM2_ESM.pdf]

Supplementary materials

Figure S1. Description of the prime, ALA linker, structural alphabet linker and structural alphabet superposition linker methods. The figure shows 22 of the 34 new methods included in PEPOP 2.0. From the 3D structure of the protein, a region is targeted in which a reference segment starts the peptide sequence. This is further elongated by using the closest neighbor segments. The successive addition of segments is marked with the rainbow colors (from violet to red); amino acid linkers are in black. For example, the NN method started with S40, followed by S61, S41, S47, S48 and finally S39, leading to the sequence `IEIKQTQNSTWFNNNK`. From this prime method, the NNsas method will give `IREIRKQLTQTNSTWFNNNK.`

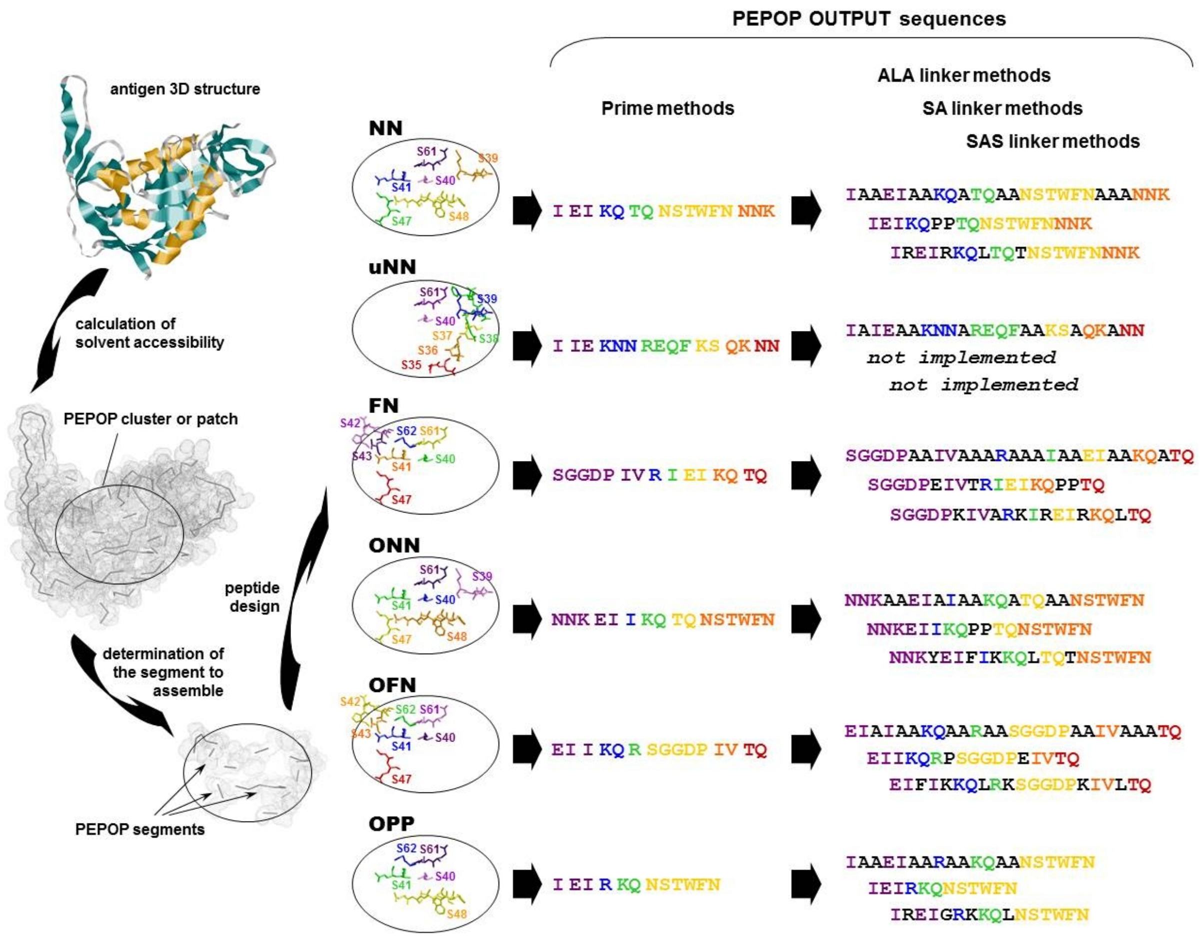

Figure S2. Sequence redundancy between peptides predicted by the various PEPOP methods. (A) The matrix illustrates the comparison between sets of peptides predicted by each method, from black (the peptides of the two sets are identical) to white (the peptides of the two sets have less than 10% identity) (B) Dendrogram of the matrix of the methods' similarity.

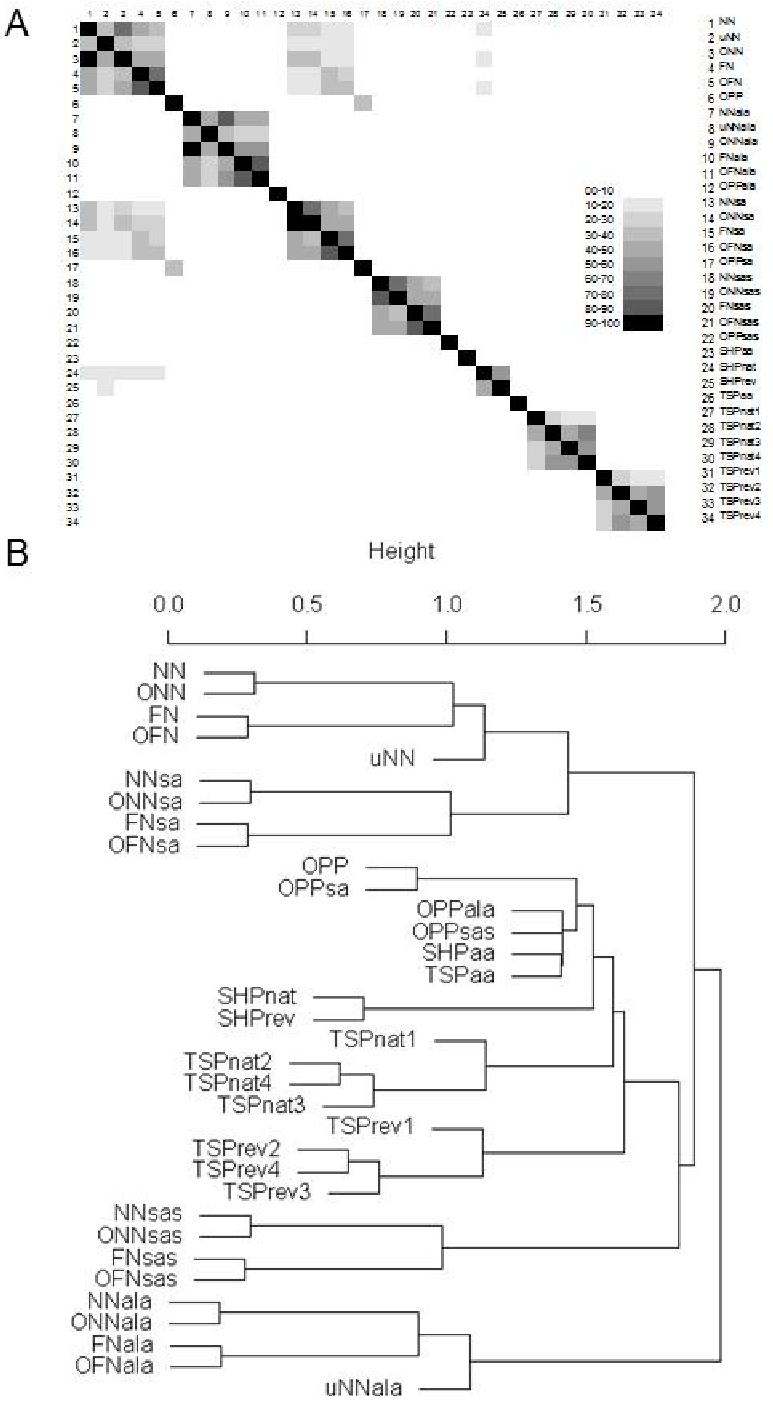

Figure S3. Characterization of PEPOP clusters and patches. (A) Number of clusters and patches according to the antigen size with (✸) the segments, the 10Å-radius patches and the 15Å-radius patches, (▲) the clusters and (■) the varying patches; (B) Histogram of the distribution (in %) of PEPOP clusters and patches including the highest number of epitopic aa; (C) distribution (in %) of PEPOP clusters and patches including the highest number of epitopic aa according to the antigen size; in (B) and (C): empty bars/symbols: without taking into account the aa positions and filled bars/symbols: by taking into account the aa positions.

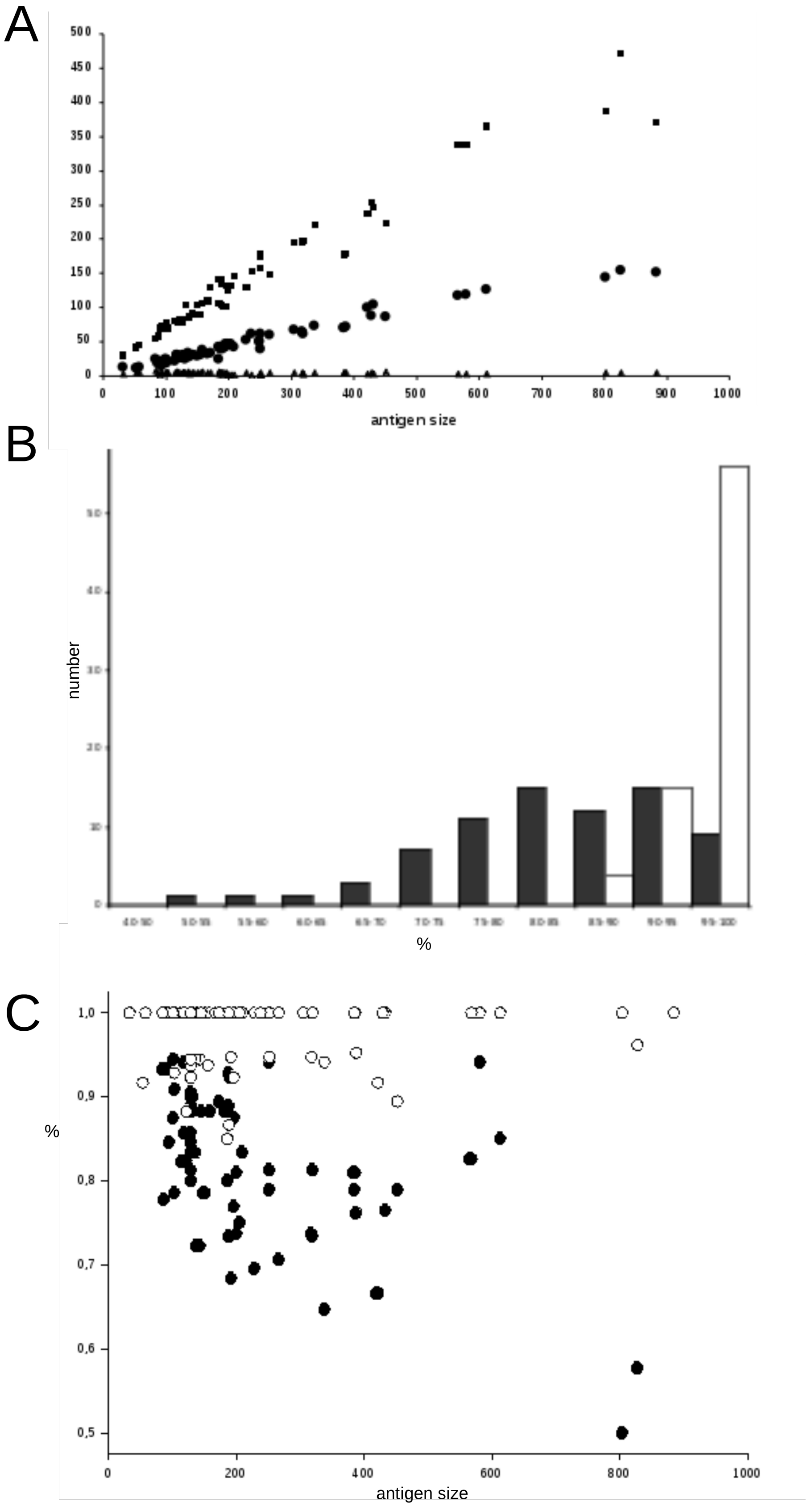
A
500
450
400
350
300
250
200
150
100
50
0
0 100 200 300 400 500 600 700 800 900 1000
antigen size
B
number
%
C
1,0
0,9
0,8
0,7
0,6
0,5
%
0 200 400 600 800 1000
antigen size

Figure S4. Mean Se (A) and PPV (B) by method. Empty bars: results that did not take into account the aa positions; filled bars: results taking into account the aa positions.

The PEPOP methods have different profiles in the graph due to their algorithm. All the prime methods have a similar mean PPV (about 0.55). The ALA methods are weaker by 0.15: this is due to the addition of alanine residues between segments. Actually, the ALA methods increase the number of FP, whereas the number of well predicted aa (TP) is roughly the same. The mean PPV values for the two other linker methods (SA and SAS methods) are lower than the PPV of the prime methods, but higher than those of the ALA methods. The aa added by these linker methods decrease the PPV by increasing the number of FP, as observed for the ALA methods. However, the aa added by the SA(S) methods might, more often, correspond to an epitope residue than the alanine added in the ALA methods, and thus fall into TP instead of FP. The mean PPV of SAS methods is lower than that of the SA methods because the SAS methods add only one aa between segments whereas, the SA methods can add between zero and two different aa, thus increasing the chance of matching an epitope residue. The mean PPV values of the graph-based methods are similar. The OPP method had the best mean PPV value.

The profile of the mean Se values is different. Taking the prime methods as reference, the mean Se values of the ALA, SA and SAS methods are progressively increasing. Differently from the mean PPV values, the aa added between the segments improve the mean Se values because they can potentially correspond to an epitope residue (TP) and the FP is not taken into account. The OPP methods have mean Se values slightly lower (by about 0.1), because these methods lead to small peptides and consequently to fewer aa than those present in an average epitope. The SHP methods also have slightly lower mean Se values than the other methods for the same reason. TSP-based methods and SAS methods have the highest mean Se values.

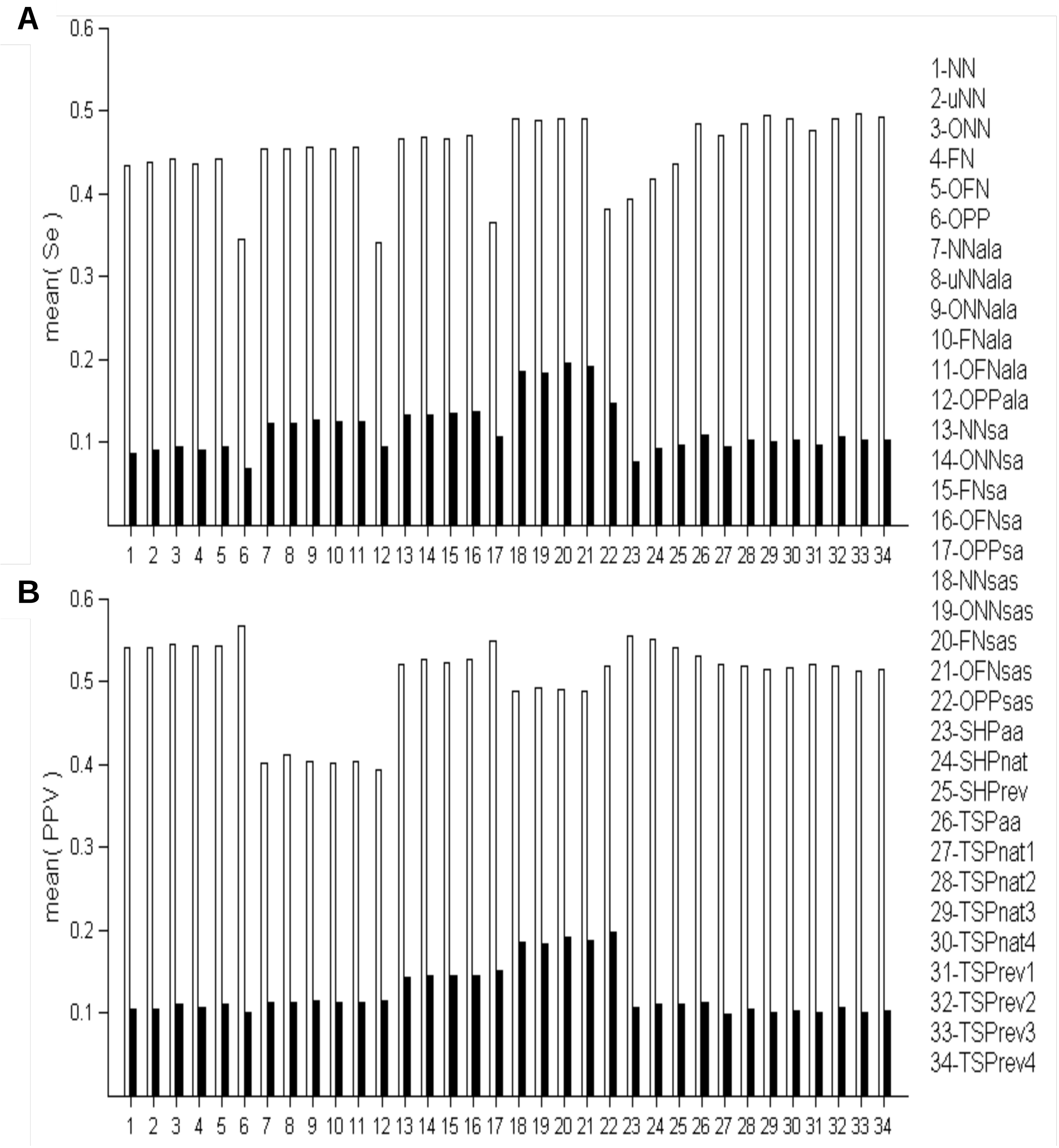
A
B
mean(Se)
mean(PPV)
0.6
0.5
0.4
0.3
0.2
0.1
1 2 3 4 5 6 7 8 9 10 11 12 13 14 15 16 17 18 19 20 21 22 23 24 25 26 27 28 29 30 31 32 33 34
1-NN
2-uNN
3-ONN
4-FN
5-OFN
6-OPP
7-NNala
8-uNNala
9-ONNala
10-FNala
11-OFNala
12-OPPala
13-NNsa
14-ONNsa
15-FNsa
16-OFNsa
17-OPPsa
18-NNsas
19-ONNsas
20-FNsas
21-OFNsas
22-OPPsas
23-SHPaa
24-SHPnat
25-SHPrev
26-TSPaa
27-TSPnat1
28-TSPnat2
29-TSPnat3
30-TSPnat4
31-TSPrev1
32-TSPrev2
33-TSPrev3
34-TSPrev4

Figure S5.

Se and PPV distribution without taking into account the positions of the peptides predicted by A) the NN methods (NN, NNala, NNsa, NNsas, uNN); B) the FN methods (FN, FNala, FNsa, FNsas); C) the ONN methods (ONN, ONNala, ONNsa, ONNsas); D) the OPP methods (OPP, OPPala, OPPsa, OPPsas); E) the SHP methods (SHPnat, SHPrev, SHPaa); and F) the TSP methods (TSPnat1, TSPnat2, TSPnat3, TSPnat4, TSPrev1, TSPrev2, TSPrev3, TSPrev4, TSPaa).

The NN, ONN, and FN methods produced about 12% of peptides with a Se of at least 0.6 and about 1.4% of peptides with a Se of 0.8. The SHP and OPP methods showed the weakest percentage (11%) of peptides with a Se of 0.6 and the TSP method the highest proportion (22%). The threshold of 0.8 was not appropriate because the percentages were too low. Whatever the method, about 25% of peptides had a PPV of at least 0.6. According to the number of peptides predicted on average per method (3508), the threshold of 0.6 is not appropriate because the percentages are too high. Combining the results from the Se and PPV analyses, the most appropriate threshold is 0.7.

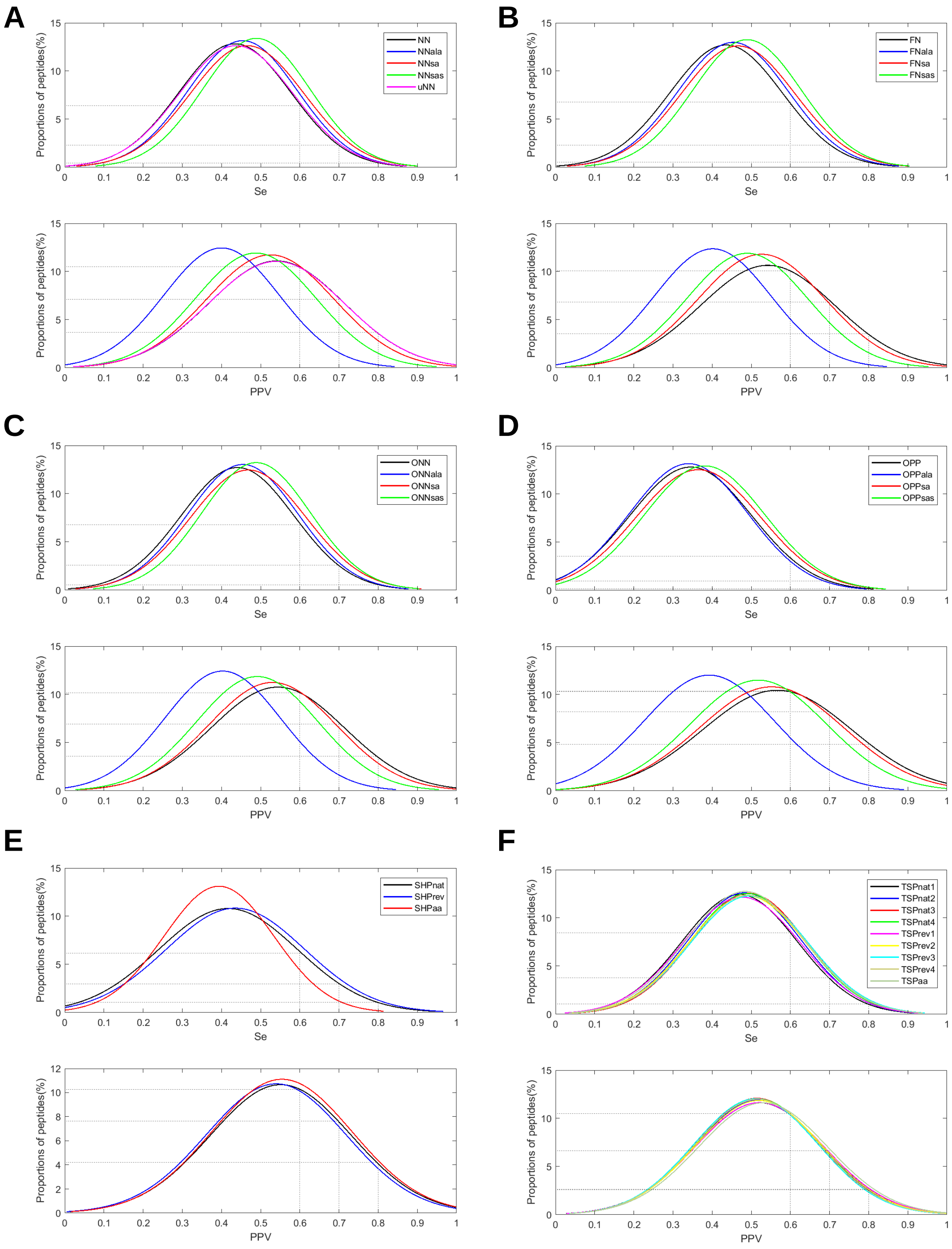
A
B
C
D
E
F
Proportions of peptides(%)
Se
PPV
NN
NNala
NNsa
NNsas
uNN
FN
FNala
FNsa
FNsas
ONN
ONNala
ONNsa
ONNsas
OPP
OPPala
OPPsa
OPPsas
SHPnat
SHPrev
SHPaa
TSPnat1
TSPnat2
TSPnat3
TSPnat4
TSPrev1
TSPrev2
TSPrev3
TSPrev4
TSPaa

Discussion

For benchmarking the PEPOP methods, we first chose not to take into account the aa positions because we think that for the antibody is not important where a key aa is in the protein as long as it is present in the peptide (in the suitable arrangement/disposition). However, taking into account the aa positions allows slightly measuring the contiguity of the aa and the segment and the aa order (which has not been made by chance). This notion is very important for antibody recognition and was considered in the PEPOP methods, but could not be assessed. Therefore, we verified whether performances were confirmed also when taking into account the aa positions.

The weaker performance of the random method proves the relevance of the PEPOP methods. In the calculations that do not take into account the aa positions, the gap between the random method, which is not present in the graphs of Figure 4 and 5, and the PEPOP methods shows even better that peptides were not predicted by chance. It also compensates a little for the lack of a method to evaluate the relevance of the aa order in the peptide compared to the epitope in order to say whether a peptide, compared to another one, may have more chances to be recognized by an antibody because the aa order in such peptide better mimics the aa order in the epitope.

Benchmarking different computational methods must be taken with precaution as from one analysis to the other, the tools, datasets and metrics can be different, thus not allowing objective, direct comparisons. These drawbacks have encouraged immunologists and bioinformaticians to discuss the current state and the future of epitope prediction and to develop recommendations for the research community [1]. We paid close attention to these recommendations. However, by developing PEPOP, we tackle the problem by a different angle because PEPOP does not predict epitopes, but peptides representative of epitopes.

Therefore, we need an experimental or theoretical procedure that can evaluate methods for predicting "discontinuous" peptides, such as the PEPOP or SUPERFICIAL methods. Such a procedure should allow the objective evaluation of how the goal of a method is achieved. It will, for example in the case of the PEPOP linker methods, demonstrate (or refute) the importance of spacing the segments in the peptide as in the epitope. This will be very informative for understanding the rules of the molecular mimicry, a very difficult [2–4], but promising research field.

References

1. Greenbaum JA, Haste Andersen P, Blythe M, Bui HH, Cachau RE, Crowe J, et al. Towards a consensus on datasets and evaluation metrics for developing B-cell epitope prediction tools. J Mol Recognit. 2007;20:75–82.

2. Van Regenmortel MH. Reductionism and complexity in molecular biology. Scientists now have the tools to unravel biological and overcome the limitations of reductionism. EMBO Rep. 2004;5:1016–20.

3. Irving MB, Craig L, Menendez A, Gangadhar BP, Montero M, van Houten NE, et al. Exploring peptide mimics for the production of antibodies against discontinuous protein epitopes. Mol Immunol. 2010;47:1137–48.

4. Chen SW, Van Regenmortel MH, Pellequer JL. Structure-activity relationships in peptide-antibody complexes: implications for epitope prediction and development of synthetic peptide vaccines. Curr Med Chem. 2009;16:953–64.
